# Potent and persistent antibody responses against the receptor-binding domain of SARS-CoV spike protein in recovered patients

**DOI:** 10.1186/1743-422X-7-299

**Published:** 2010-11-04

**Authors:** Zhiliang Cao, Lifeng Liu, Lanying Du, Chao Zhang, Shibo Jiang, Taisheng Li, Yuxian He

**Affiliations:** 1 State Key Laboratory for Molecular Virology and Genetic Engineering, Institute of Pathogen Biology, Chinese Academy of Medical Sciences and Peking Union Medical College, Beijing 100730, China; 2 Peking Union Medical college Hospital, Chinese Academy of Medical Sciences and Peking Union Medical College, Beijing 100730, China; 3 Lindsley F. Kimball Research Institute, New York Blood Center, New York, NY10065, USA

## Abstract

**Background:**

The spike (S) protein of SARS-CoV not only mediates receptor-binding but also induces neutralizing antibodies. We previously identified the receptor-binding domain (RBD) of S protein as a major target of neutralizing antibodies in animal models and thus proposed a RBD-based vaccine. However, the antigenicity and immunogenicity of RBD in humans need to be characterized.

**Results:**

Two panels of serum samples from recovered SARS patients were included and the antibody responses against the RBD were measured by ELISA and micro-neutralization assays. We found that the RBD of S protein induced potent antibody responses in the recovered SARS patients and RBD-specific antibodies could persist at high titers over three year follow-up. Furthermore, affinity purified anti-RBD antibodies possessed robust neutralizing activity.

**Conclusion:**

The RBD of SARS-CoV is highly immunogenic in humans and mediates protective responses and RBD-based vaccines and diagnostic approaches can be further developed.

## Background

The global outbreak of severe acute respiratory syndrome (SARS), caused by a novel coronavirus (SARS-CoV), resulted in more than 8,000 cases with a fatality rate of about 10%. Impressively, the rapid spread of SARS-CoV made a great impact on public health and social-economic stability. It is thought that SARS-CoV might originate from its natural reservoir bats and transmit to humans through an intermediate such as palm civets and raccoon dogs, and no one can exclude the possibility of its recurrence [1].

SARS-CoV is an enveloped positive-stranded RNA virus and its "crown"-like spike (S) protein has two major biological functions: 1) mediating receptor (angiotensin converting enzyme 2, ACE2) binding and membrane fusion; 2) inducing neutralizing antibody responses [2,3]. The S protein was considered as an important target for developing diagnostics, vaccines and therapeutics [4-12]. The receptor-binding domain (RBD) of S protein was defined as a fragment corresponding to the residues 318 - 510 of the S protein, which mediates viral binding to cell receptor ACE2 [13-15]. Coincidently, we identified the RBD as a major target of neutralizing antibodies [16-19], and proposed it as an ideal vaccine antigen for clinical application [20-22]. The immunogenicity and protective efficacy of RBD-based vaccine candidates have been evaluated in animal models [17,23-25]. However, the antigenicity and immunogenicity of RBD in humans need to be characterized in detail toward developing the RBD-based vaccines and diagnostics. In this short communication, we found that patients recovered from SARS developed potent and persistent RBD-specific antibody responses, highlighting the potentials of clinical applications of RBD-based vaccines and diagnostics.

## Materials and methods

### Serum samples from SARS patients

Two panels of serum samples from the recovered SARS patients were used in this study. The first panel of 35 samples were leftover from the previous study [12], which were collected from the convalescent-phase SARS patients 30-60 days after onset of illness during the 2003 outbreak in Beijing. The second panel of sequential samples were collected from 19 SARS patients, who were enrolled in March 2003 for a follow-up study at the Peking Union Medical College Hospital, Beijing. All patients were diagnosed as SARS according to the criteria released by WHO and verified to be serologically positive by clinical laboratories. Informed consent was obtained from each participant.

### Expression of recombinant RBD proteins

The RBD-His (RBD sequence with a His-tag) and RBD-Fc (RBD fused with human IgG-Fc) proteins were respectively expressed and purified as described previously [16,23]. In brief, the plasmid encoding RBD-His or RBD-Fc was transfected into HEK293T cells using Lipofectamine 2000 (Invitrogen, Carlsbad, CA) according to the manufacturer's protocols. Culture medium was replaced by fresh OPTI-MEM I Reduced-Serum Medium 12 h post-transfection and the supernatants containing expressing RBD proteins were collected 72 h later. RBD-His was purified by Nickel affinity column (Qiagen), while RBD-Fc was purified by protein A-Sepharose 4 Fast Flow (Amersham Biosciences, Piscataway, NJ).

### ELISA

The reactivity of SARS serum samples or purified anti-RBD antibodies with recombinant RBD protein was determined by ELISA. Briefly, 1 μg/ml purified RBD-His was coated onto wells of 96-well microtiter plates (Corning Costar, Acton, MA) in 0.1 M carbonate buffer (pH 9.6) at 4°C overnight. After blocking with 5% non-fat milk for 2 h at 37°C, diluted samples were added and incubated at 37°C for 1 h, followed by three washes with PBS containing 0.1% Tween 20. Bound antibodies were detected with HRP-conjugated goat anti-human IgG (Invitrogen, Carlsbad, CA) at 37°C for 1 h, followed by three washes. The reaction was visualized by addition of the substrate 3,3',5,5'-tetramethylbenzidine (TMB) and stopped by addition of 2N H_2_SO_4_. Absorbance at 450 nm was measured by ELISA Microplate Reader (Bio-Rad, Hercules, CA). Total serum IgG antibodies against SARS-CoV were measured using commercially available whole virus lysates-based ELISA kits (BJI-GBI Biotechnology, Beijing, China).

### Immunoaffinity chromatography

The immunoaffinity resin for the purification of RBD-specific antibodies was prepared as described previously [19]. In brief, the RBD-Fc fusion protein was coupled to cyanogenbromide-activated Sepharose beads (Pharmacia, Piscataway, NJ) according to the manufacturer's instruction. For immunoadsorption, patient serum sample was diluted 10-fold with PBS and incubated with the RBD-Fc resin overnight at 4°C with constant rotation. Resin was then packed into a 5-ml column and the flowthrough was discarded. After the resin was washed with 10× column volumes of PBS, the bound antibodies (anti-RBD) were eluted in 0.2 M glycine-HCl buffer, pH 2.5. The eluates were immediately neutralized with Tris buffer (pH 9.0). Then, the buffer was exchanged with PBS by several cycles of dilution and concentrated by Amicon Ultra-15 centrifugal filter device (Millipore Corporation, Bedford, MA). The purified anti-RBD antibodies were sterilized with 0.2-μm pore size microspin filters (Millipore) and the concentrations were measured.

### Micro-neutralization assays

SARS pseudovirus system was developed in our laboratory as previously described. In brief, HEK293T cells were co-transfected with a plasmid encoding codon-optimized SARS-CoV S protein (Tor2) and a plasmid encoding Env-defective, luciferase expressing HIV-1 genome (pNL4-3.luc.RE) using Lipofectamine™ 2000 reagents (Invitrogen) according to the manufacturer's protocol. Supernatants containing pseudovirus bearing the S protein were harvested 48 h post-transfection and used for single-cycle infection of ACE2-expressing 293T cells (293T/ACE2). Briefly, 293T/ACE2 cells were plated (10^4 ^cells/well) in 96-well tissue culture plates and grown overnight. The pseudovirus was preincubated with serially diluted purified anti-RBD at 37°C for 1 h before addition to cells. The culture was re-fed with fresh medium 12 h later and incubated for an additional 48 h. Cells were washed with PBS and lysed using lysis reagent included in the luciferase kit (Promega, Madison, WI). Aliquots of cell lysates were transferred to 96-well Costar flat-bottom luminometer plates (Corning Costar, Corning, NY), followed by addition of luciferase substrate (Promega). Relative light units (RLU) were determined immediately on the Modulus™ II Microplate Multimode Reader (Turner Biosystems, Sunnyvale, CA).

## Results

### Potent RBD-specific antibody responses in SARS-CoV infected patients

**Figure 1:**
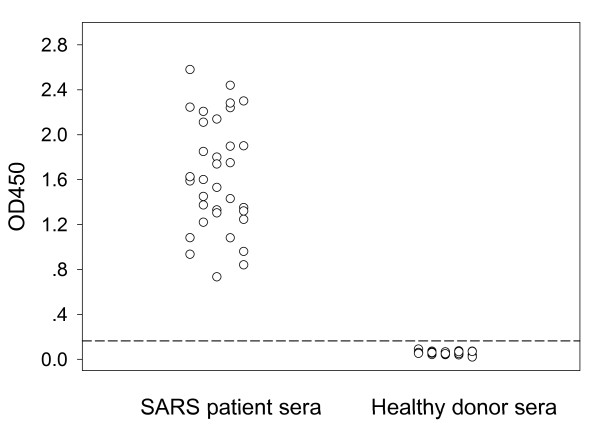
**Potent RBD-specific antibody responses in the recovered SARS patients**. The convalescent sera from 30 SARS patients and normal sera from 25 healthy blood donors were tested at 1/100 dilution by ELISA with RBD-His protein as a coating antigen. The dashed line represents a cutoff value (the mean absorbance at 450 nm of sera from healthy blood donors plus 3 SDs).

Because our previous studies indicated that the RBD of S protein was a major target of neutralizing antibodies and that the RBD-based immunogens could induce potent neutralizing responses and protective immunity in animal models, we intended to know the immunogenicity of RBD in infected patients. A panel of 35 convalescent sera collected from the SARS patients 30 - 60 days after onset of illness were previously used for mapping the antigenic sites of SARS-CoV [12]. The leftover samples were stored in -80°C and expected to maintain their activities to interact with SARS-CoV antigens. Therefore, we used the purified RBD-His protein as a coating antigen in ELISA to detect RBD-specific IgG antibodies in these SARS serum samples. As shown in Figure 1, all 35 convalescent sera at 100-fold dilutions significantly reacted with the RBD-His protein with an average OD_450 _value of 1.63. As a control, none of the sera from healthy blood donors was reactive to this antigen. The results demonstrated that the S protein RBD is highly immunogenic in the SARS-CoV infected patients.

### Persistent RBD-specific antibody responses in SARS-CoV infected patients

**Figure 2:**
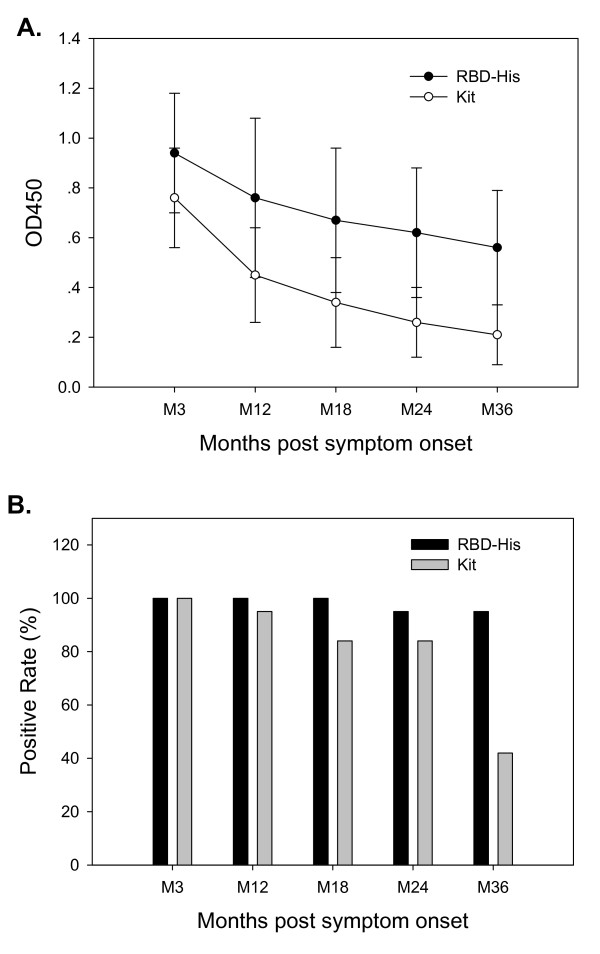
**Persistent RBD-specific antibody responses in the recovered SARS patients**. The sequential samples were collected from 19 recovered SARS patients enrolled for a follow-up study. The sera were tested at 1/100 dilution by ELISA with RBD-His protein as a coating antigen or by the viral lysate-based diagnostic kit.

To study the antigenicity and immunogenicity of SARS-CoV in humans, we enrolled a cohort during the SARS outbreak in 2003, and 19 patients were followed-up over three years. The sequential blood samples were collected at month 3, 12, 18, 24, and 36, respectively, after the onset of clinical symptom. All serum samples were tested at 100-fold dilutions for RBD-specific IgG antibodies by ELISA with RBD-His as a coating antigen. In a parallel, total serum IgG antibodies specific for SARS-CoV were also detected using the commercial ELISA kit, in which the whole virus lysates were coated as antigen. The serum samples were diluted at 1:10 according to the manufacturer's instruction. The results are presented in Figure 2. The RBD-specific antibodies maintain relatively higher titers through 3 year follow-up. At year 2 and year 3, only one same sample became undetectable and thus gave a positive rate of 94.74%. However, the ELISA results from the kit showed much low reactivity. Compared to the RBD-based results, the OD values for all samples dropped dramatically at year 2 and 3. Specially, the positive percentage of the year 3 samples was only 42.11% (8/19), suggesting the viral lysate-based ELISA kit had much low sensitivity than the RBD-based ELISA.

### RBD-specific human antibodies possess potent neutralizing activity

**Figure 3:**
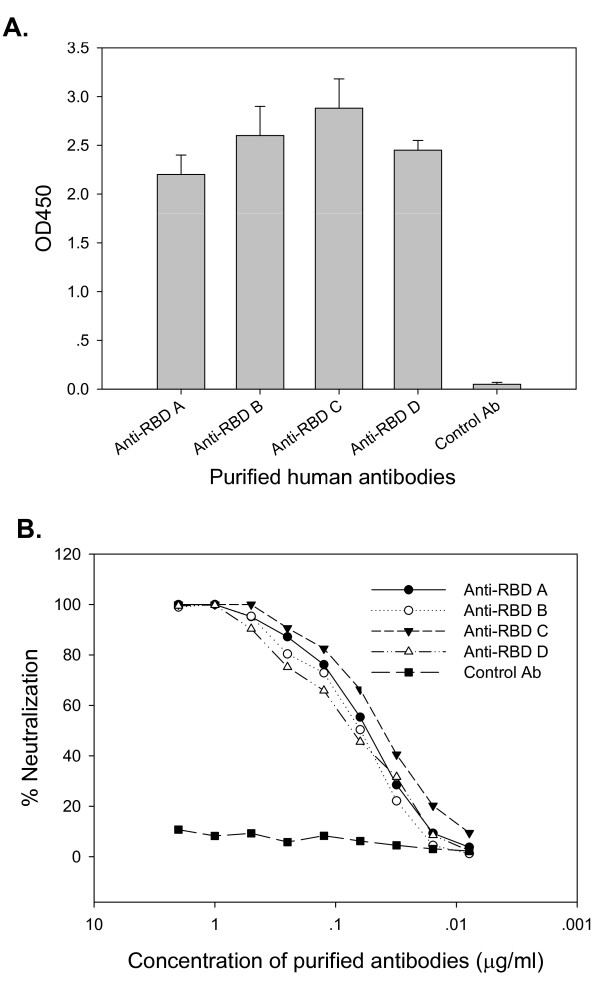
**Neutralization of SARS pseudovirus infection by affinity-purified RBD-specific antibodies from the recovered SARS patients**. Infection of 293T/ACE2 cells by SARS pseudovirus was determined in the presence of RBD-specific antibodies at a series of 2-fold dilutions, and percent neutralization was calculated for each sample.

We and others previously reported that RBD-specific polyclonal and monoclonal antibodies had potent neutralizing activity against SARS-CoV infection *in vitro *and *in vivo *[16,17,26,27]. We have kept asking whether RBD-specific antibodies are responsible for serum-mediated neutralizing activity. To this end, we purified RBD-specific antibodies by immunoaffinity chromatography from four SARS serum samples selected from the convalescent-phase SARS patients 30-60 days after onset of illness during the 2003 Beijing outbreak. The fusion protein RBD-Fc was coupled to cyanogenbromide-activated Sepharose beads and the serum sample was flowed through the bead column to absorb the RBD-specific antibodies. Figure 3A shows that the purified RBD-specific antibodies from SARS patients strongly reacted with RBD-His antigen. Then, we tested their neutralizing activity against SARS pseudoviruses. Consistently, all the four purified human RBD-specific antibodies potently neutralized infection by SARS pseudoviruses, with an average ND_50 _of 0.05 μg/ml (Figure 3B).

## Discussion

The sudden emergence of SARS-CoV shocked the world and impacted the public health seriously. The pandemic was contained under high level quarantines, but its disappearance also raised numerous mysteries to the research community. Even today we know little about its severe pathogenesis and have no effective treatment protocols. In preparedness, we also need effective diagnostic approaches and preventive vaccines.

One of our previous major findings is that the RBD of S protein serves as a main antigenic site that mediates neutralizing antibody responses [12,16-18,28-33]. We have clearly demonstrated that the RBD contains multiple conformation-dependent neutralizing epitopes [16,18] and that RBD-based immunogens can induce potent neutralizing antibodies and protective immunity [16,17,23-25,29,30,34]. Based on these observations we have proposed RBD-based SARS vaccines [20-22]. However, our previous studies were primarily based on the animal models. Toward developing a safe and effective RBD-based vaccine one fundamental question is that whether the RBD is immunogenic and functional for neutralizing antibodies in humans. In our ongoing preclinical studies, the immunogenicity of RBD immunogens will be evaluated in non-human primates. In the present study, we firstly investigated the RBD-specific antibody responses in subjects recovered from SARS-CoV infection as an alternative way to evaluate RBD vaccine in humans. With a recombinant RBD protein as an ELISA antigen, we showed that the spike RBD could induce potent and persistent antibody responses in the recovered patients (Figure 1 and 2); with a RBD-Fc fusion protein in the immunoaffinity column, the anti-RBD antibodies purified from the positive human serum samples possessed potent neutralizing activity (Figure 3). Therefore, these data verify the functionality of SARS-CoV RBD in infected subjects and highlight RBD-based vaccines for human application.

The antibody detection is critical for serodiagnosis of SARS-CoV infection, but the sensitivity and specificity of current approaches need to be improved. Although a number of antigens, including proteins or peptides, have been proposed for SARS-CoV serodiagnosis, the first and only serological diagnostic kit in China is based on the viral lysates-derived antigen. Our results also demonstrated that the lysate-based ELISA kit showed much lower reactivity with human positive sera while compared to the RBD-based ELISA (Figure 2). We were not surprised by the kit-based results since similar serological data were previously reported [35,36]. By using the same brand kit in a follow-up study, Wu *et al *reported that the average OD values dropped to 0.25 by year 3 and gave a positive rate of 55.56% [35], consistent with the present results. Therefore, the clinical application of the viral lysate-based ELISA kit might be limited by its sensitivity and specificity, and development of novel improved approaches for SARS-CoV serodiagnosis remains to be a priority. The S protein RBD-based methods can be further explored as the RBD specifically mediates receptor-binding and is a major antigenic sites in both animals and humans.

## Competing interests

The authors declare that they have no competing interests.

## Authors' contributions

ZLC, LFL, LYD and CZ mainly carried out the experiments and data analysis. SBJ, TSL and YXH conceived the studies and wrote the manuscript. All authors read and approved the final manuscript.
